# Risk prediction for long-term cardiovascular events in patients with concurrent hypertension, HFpEF, and unstable angina: a multicenter machine learning-assisted cohort study

**DOI:** 10.3389/fendo.2026.1851201

**Published:** 2026-07-23

**Authors:** Long Tang, Xiaomeng Yang, Zongchao Zuo, Jiajun Zhu, Wenyuan Yin, Bowen Zhou, Tongjian Zhu, Bing Wu, Hui Li, Jun Wang, Shoupeng Duan

**Affiliations:** 1 Department of Cardiology, The Affiliated Xuancheng Hospital of Wannan Medical University, Xuancheng, Anhui, China; 2 Shenzhen Hospital, Southern Medical University, Shenzhen, Guangdong, China; 3 Department of Cardiology, The First Affiliated Hospital of Bengbu Medical University, Bengbu, Anhui, China; 4 Cardiac Care Unit, First Affiliated Hospital of Xinjiang Medical University, Urumchi, China; 5 Electrocardiology Department, People’s Hospital of Xinjiang Uygur Autonomous Region, Urumqi, China; 6 Department of Cardiology, Suzhou First People’s Hospital, Suzhou, Anhui, China; 7 Department of Cardiology, Xiangyang Central Hospital, Xiangyang, Hubei, China; 8 Joint Research Center for Regional Diseases of IHM,Bengbu Medical University, Bengbu, Anhui, China; 9 Joint Research Center for Regional Diseases of IHM, The First Affiliated Hospital of Bengbu Medical University, Bengbu, Anhui, China; 10 Department of Cardiology, Renmin Hospital of Wuhan University, Wuhan, Hubei, China; 11 Department of Cardiology, Wuhan No.1 Hospital, Wuhan, China; 12 Institute of Clinical Medicine and Department of Cardiology, Renmin Hospital, Hubei University of Medicine, Shiyan, Hubei, China

**Keywords:** heart failure with preserved ejection fraction (HFpEF), hypertension, machine learning, prediction nomogram, unstable angina pectoris

## Abstract

**Background:**

Patients with concurrent hypertension, heart failure with preserved ejection fraction (HFpEF), and unstable angina pectoris (UAP) represent a high-risk population with heterogeneous long-term cardiovascular outcomes. However, practical tools for individualized risk stratification in this clinically complex population remain limited. This study aimed to develop and validate a machine learning-assisted nomogram for predicting long-term major adverse cardiovascular events (MACEs) in patients with concomitant hypertension, HFpEF, and UAP.

**Methods:**

This multicenter retrospective cohort study included 1,669 patients with hypertension, HFpEF, and UAP from Six medical centers in China. Patients were assigned according to treating hospital to a development cohort, a validation cohort, and an independent test cohort. The primary endpoint was MACEs, defined as cardiac death, unplanned coronary revascularization, or rehospitalization for acute heart failure. Candidate predictors were selected using least absolute shrinkage and selection operator regression, the Boruta algorithm, and random forest feature importance analysis. Variables consistently identified across these methods were entered into a multivariable Cox proportional hazards model, and a Cox-based nomogram was constructed to estimate 2-, 3-, and 4-year MACE risk. Model performance was assessed using receiver operating characteristic curves, time-dependent area under the curve, calibration curves, decision curve analysis, and Kaplan-Meier survival analysis.

**Results:**

During a median follow-up of 48 months, 254 patients experienced MACEs. Five predictors were retained in the final model: diabetes mellitus, previous myocardial infarction, systemic immune-inflammation index, triglyceride, and N-terminal pro-B-type natriuretic peptide. In the development cohort, the nomogram yielded area under the curve values of 0.785, 0.787, and 0.760 for predicting 2-, 3-, and 4-year MACEs, respectively. The corresponding values were 0.674, 0.697, and 0.682 in the validation cohort, and 0.685, 0.711, and 0.718 in the test cohort. Calibration curves showed acceptable agreement between predicted and observed risks, and decision curve analysis suggested potential net benefit across clinically relevant threshold probabilities. Patients classified as high risk according to the nomogram had a significantly higher incidence of MACEs than those classified as low risk across all cohorts.

**Conclusion:**

A machine learning-assisted, Cox-based nomogram incorporating five routinely available clinical and laboratory variables provided acceptable discrimination in the development cohort and modest-to-acceptable performance in geographically distinct validation and test cohorts. This model may support individualized long-term risk stratification in patients with concurrent hypertension, HFpEF, and UAP, although prospective validation and clinical impact studies are warranted before routine implementation.

## Graphical Abstract

**Figure:**
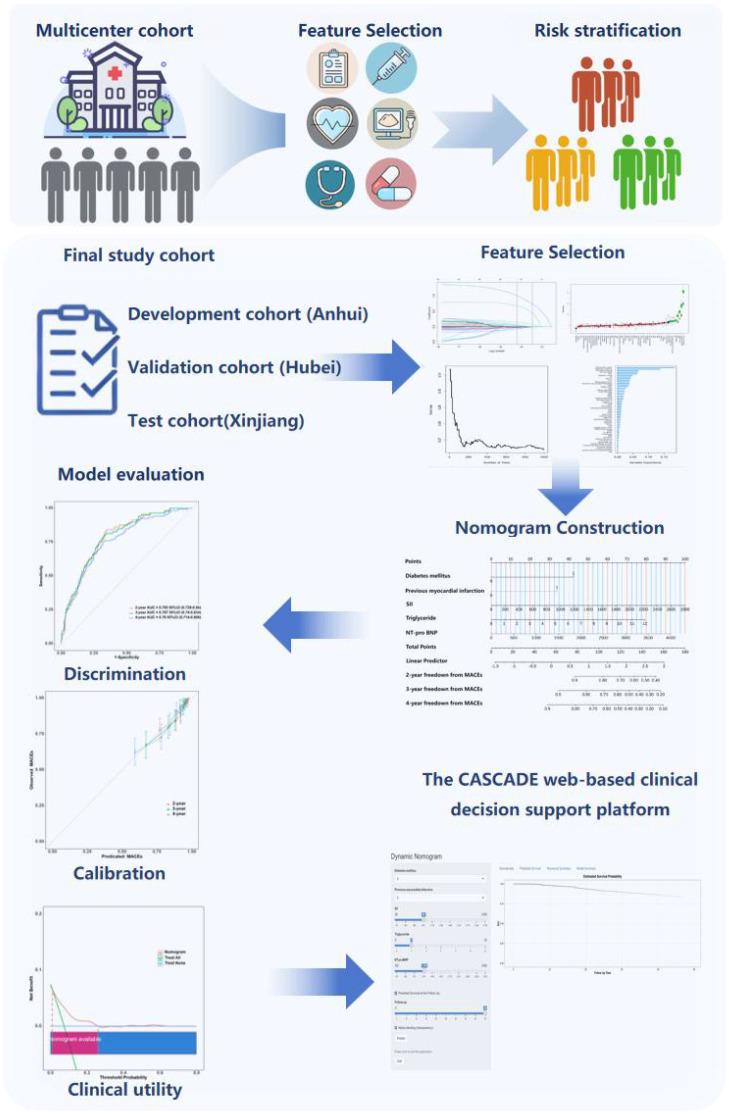

## Introduction

Hypertension, heart failure with preserved ejection fraction (HFpEF), and unstable angina pectoris (UAP) are highly prevalent cardiovascular disorders associated with substantial morbidity and mortality (1–4). Globally, approximately 1.28 billion adults aged 30–79 years have hypertension, and China has more than 240 million affected adults (1). Heart failure affects more than 60 million people worldwide, with HFpEF accounting for nearly one half of all heart failure cases, particularly among older patients with hypertension and metabolic comorbidities (2). UAP remains a major presentation within the acute coronary syndrome spectrum and contributes substantially to coronary hospitalizations in real-world clinical practice (3). However, despite the high prevalence of these individual conditions, epidemiological data on the combined prevalence of concurrent hypertension, HFpEF, and UAP remain scarce.

Although concurrent hypertension, HFpEF, and UAP are traditionally recognized and managed as separate nosological entities, accumulating mechanistic evidence suggests that their frequent co-occurrence may reflect shared pathophysiological processes rather than coincidental comorbidity (4–8). This cardiovascular triad can be conceptualized as a complex disease network in which persistent hypertension promotes maladaptive myocardial remodeling and ultimately contributes to HFpEF (1, 2, 4, 5). In parallel, systemic inflammatory activation and cardiometabolic dysregulation accelerate atherosclerotic disease activity and increase coronary plaque vulnerability, thereby predisposing patients to UAP (2, 3, 7, 8).

Notably, these conditions often co-occur in real-world clinical practice, and the resulting multimorbidity profile may be more clinically consequential than any single diagnosis in isolation (1–8). In current practice, disease-specific management pathways frequently run in parallel: antihypertensive therapy primarily targets blood pressure control, HFpEF management emphasizes symptom-driven hemodynamic optimization, and UAP requires intensive anti-ischemic and antithrombotic strategies (1–3). However, the combined clinical impact of this hypertension-HFpEF-UAP phenotype remains incompletely characterized, and existing prognostic tools do not provide a sufficiently integrated framework for individualized long-term risk assessment and follow-up decision-making. Moreover, many available prediction models are based on linear statistical assumptions and fixed thresholds, limiting their ability to capture the high-dimensional, interacting metabolic and inflammatory signals that plausibly drive risk in multimorbid patients (6, 9, 10).

Given their capacity to identify complex nonlinear patterns and high-dimensional interactions from clinical data, machine learning approaches offer a promising strategy to address these limitations (11). Beyond traditional regression-based approaches, an increasing body of cardiovascular studies has leveraged machine learning to derive prognostic models from high-dimensional clinical data. For example, data-driven multimodal frameworks have been developed to identify early prognostic markers using multimodal visualization and explainable machine learning in patients with HFpEF and UAP (6). Similar machine learning-based multimodal risk prediction models have also been applied to predicting poor outcomes in post-transcatheter aortic valve replacement heart failure hospitalizations among patients with concurrent symptomatic aortic stenosis and HFpEF. Collectively, these studies support the feasibility and clinical relevance of machine learning pipelines-including high-dimensional feature screening and model construction-for improving long-term cardiovascular risk stratification (6, 7, 9, 10).

Although prognostic models have previously been developed for patients with UA and HFpEF (6), limited evidence is available for the more specific and clinically common subgroup of patients who also have hypertension. Given the pathophysiological links between hypertension, diastolic dysfunction, vascular stiffness, endothelial dysfunction, and coronary ischemia, this subgroup may have a distinct risk profile that warrants dedicated evaluation. Therefore, this multicenter retrospective cohort study aimed to develop and validate a machine learning-assisted, Cox-based nomogram for predicting long-term MACEs in patients with concurrent hypertension, HFpEF, and unstable angina pectoris.

## Methods

### Study design and participants

This retrospective multicenter CASCADE cohort study included clinical medical information of 1669 hospitalized patients with concurrent hypertension, HFpEF, and UAP. All patients were hospitalized at six hospitals in different regions of China between January 2019 and January 2020: the First Affiliated Hospital of Bengbu Medical University, Suzhou First People’s Hospital, Xiangyang Central Hospital, Renmin Hospital of Wuhan University, Renmin Hospital of Hubei University of Medicine, and First Affiliated Hospital of Xinjiang Medical University. The inclusion and exclusion criteria are presented in **Supplementary Table 1**. All included patients underwent coronary angiography during the index hospitalization, and the diagnosis of unstable angina pectoris was established based on clinical presentation, electrocardiographic findings, cardiac biomarker assessment, and coronary angiographic evaluation. Patients who underwent coronary revascularization during the index hospitalization were not included in the present analysis. This study was approved by the research ethics boards at each of the six medical centers, and the requirement for informed consent was waived due to the retrospective nature of the study.

### Data collection and laboratory tests

Demographic and clinical data, including age, gender, physical examination findings, medical history, and comorbidities, were retrieved from the electronic medical records of all participants. Upon admission, resting brachial arterial blood pressure, systolic blood pressure (SBP), and diastolic blood pressure (DBP) were recorded. Body mass index (BMI) was calculated as the ratio of body weight to the square of height. After an overnight fasting period of 8–12 h, fasting venous blood samples were collected and sent to the central laboratories of the participating medical centers for standardized biochemical and hematological analyses. Laboratory assessments included complete blood count, N-terminal pro-brain natriuretic peptide (NT-proBNP), coagulation parameters, and biochemical markers. The systemic immune-inflammation index (SII) was calculated as the platelet count multiplied by the neutrophil count and divided by the lymphocyte count, serving as an indicator of systemic inflammation and immune response (12). The systemic inflammation response index (SIRI) was determined as the neutrophil count multiplied by the monocyte count and divided by the lymphocyte count, reflecting systemic inflammation and immune dysregulation (13). All laboratory measurements were performed using standardized protocols to ensure data accuracy and consistency.

### Transthoracic echocardiography parameters

All TTE examinations were performed by experienced sonographers. The size of each atrial ventricle, left ventricular posterior wall thickness (LVPWD), left ventricular ejection fraction (LVEF), and other conventional ultrasound parameters were assessed according to the American Society of Echocardiography guidelines. LVEF was assessed using the modified biplane Simpson’s equation in both apical four-chamber and two-chamber views.

### Clinical endpoint and follow-up

All patients were followed up for an average of 48 months after discharge through outpatient visits or telephone interviews. The primary endpoint was major adverse cardiovascular events (MACEs), defined as a composite of cardiac death, unplanned coronary revascularization during follow-up after discharge, or rehospitalization for acute heart failure. The time of the first occurrence of MACEs was recorded as the study endpoint.

### Feature selection and model construction

To identify the most relevant predictors, three independent feature selection methods were used: least absolute shrinkage and selection operator (LASSO) regression, the Boruta algorithm, and random forest feature importance analysis. LASSO regression was applied with 10-fold cross-validation to shrink coefficients and retain the most informative variables (14). The Boruta algorithm, a wrapper method based on random forest, was used to eliminate irrelevant features by comparing them to shadow variables (15). Additionally, a random forest feature importance analysis was conducted to assess and rank the predictive value of each variable (16). Only variables consistently selected across all three methods were retained for subsequent modeling. These selected predictors were then entered into a multivariable Cox proportional hazards model to identify independent predictors of MACEs, and hazard ratios (HRs) with 95% confidence intervals (CIs) were estimated for each predictor. Variables were considered independent predictors if they showed a statistically significant association with MACEs in the multivariable Cox model using a two-sided P<0.05 criterion. A Cox-based nomogram was subsequently constructed to generate individualized predicted probabilities of 2-, 3-, and 4-year MACEs.

A nomogram was then constructed based on the significant variables identified in the Cox model to provide an individualized risk assessment tool for clinical application. The predictive performance of the nomogram was evaluated comprehensively. Discrimination ability was assessed using the receiver operating characteristic (ROC) curve and quantified using the area under the curve (AUC). Additionally, time-dependent AUC (TIME-AUC) was calculated at multiple time points to assess the dynamic predictive ability of the model over time. Calibration was examined using calibration curves by comparing the predicted and observed event probabilities. Clinical utility was assessed using decision curve analysis (DCA) to determine the net benefit across different threshold probabilities. To further validate the prognostic utility of the model, patients in the training, validation, and test cohorts were stratified into two groups (high- and low-risk) according to their nomogram-derived risk scores. Kaplan–Meier survival analysis was performed to compare the survival outcomes of the two groups, with differences evaluated using the log-rank test.

### Statistical analysis

All statistical analyses were performed using R (version 4.2.2). Normality of continuous variables was assessed using the Shapiro-Wilk test. Normally distributed variables were summarized as mean ± standard deviation (SD) and compared across the three cohorts using one-way analysis of variance (ANOVA), followed by Bonferroni *post hoc* tests for pairwise comparisons when appropriate. Non-normally distributed variables were summarized as median (interquartile range [IQR]) and compared using the Kruskal-Wallis test, with Dunn’s test and Bonferroni correction for *post hoc* pairwise comparisons. Categorical variables were presented as counts and percentages (n [%]) and compared using the chi-square test; Fisher’s exact test was used when any expected cell count was <5. All hypothesis tests were two-sided, and a P value <0.05 was considered statistically significant.

## Results

### Clinical characteristics

This multicenter retrospective clinical study included 1669 patients diagnosed with hypertension, HFpEF, and UAP from six medical centers in China (**Table 1**). The patients were stratified into three cohorts based on their treating hospitals: the training cohort (The First Affiliated Hospital of Bengbu Medical University and Suzhou First People’s Hospital; n = 825), the validation cohort (Renmin Hospital of Wuhan University, Renmin Hospital of Hubei University of Medicine, and Xiangyang Central Hospital; n = 473), and the test cohort (The First Affiliated Hospital of Xinjiang Medical University; n = 371). Among all enrolled patients, 952 (57.04%) were male, with a median age of 70. During a median follow-up period of 48 months, 254 patients (15.22%) experienced MACEs, including 118 (14.3%) in the development cohort, 87 (18.39%) in the validation cohort, and 49 (13.21%) in the test cohort. The incidence of MACEs was comparable among the three cohorts without statistically significant differences (*P* = 0.067).

**Table 1:** Baseline characteristics of patients in the development, validation, and test cohorts.

Characteristic and outcome	All cohort (n = 1669)	Development cohort (n = 825)	Validation cohort (n = 473)	Test cohort (n = 371)	ssP
Male sex, n (%)	952 (57.04%)	485 (58.79%)	249 (52.64%)	218 (58.76%)	0.074
Age (years)	70 (62, 76)	69 (62, 76)	70 (63, 76)	71 (64, 76)	0.282
BMI (kg/m^2^)	23.73 (22.32, 25.39)	23.74 (22.64, 25.21)	23.44 (21.72, 25.39)	24.14 (22.04, 26.44)	0.002
Diabetes mellitus, n (%)	392 (23.49%)	186 (22.55%)	121 (25.58%)	85 (22.91%)	0.443
Current smoking, n (%)	355 (21.27%)	208 (25.21%)	85 (17.97%)	62 (16.71%)	< 0.001
Current alcohol consumption, n (%)	194 (11.62%)	96 (11.64%)	62 (13.11%)	36 (9.7%)	0.309
Family history of hypertension, n (%)	105 (6.29%)	59 (7.15%)	37 (7.82%)	9 (2.43%)	0.002
Family history of CAD, n (%)	101 (6.05%)	49 (5.94%)	33 (6.98%)	19 (5.12%)	0.523
Previous myocardial infarction, n (%)	98 (5.87%)	45 (5.45%)	32 (6.77%)	21 (5.66%)	0.615
Previous PCI, n (%)	429 (25.7%)	202 (24.48%)	123 (26%)	104 (28.03%)	0.424
Previous stroke, n (%)	158 (9.47%)	74 (8.97%)	37 (7.82%)	47 (12.67%)	6.167
SBP (mmHg)	136 (125, 142)	133 (123, 141)	138 (128, 141)	138 (128, 145)	0.024
DBP (mmHg)	80 (74, 90)	80 (75, 90)	80 (73, 90)	84 (71, 95)	0.334
Heart rate (bpm)	70 (70, 78)	70 (70, 76)	72 (68, 78)	72 (70, 80)	0.003
White blood cells (10^9^/L)	6.30 (5.21, 7.67)	6.31 (5.28, 7.59)	6.18 (5.03, 7.77)	6.45 (5.19, 7.76)	0.192
Neutrophil count (10^9^/L)	3.97 (3.14, 5.11)	3.94 (3.15, 4.98)	3.88 (2.96, 5.11)	4.06 (3.3, 5.32)	0.088
Lymphocyte count (10^9^/L)	1.67 (1.26, 2.11)	1.66 (1.26, 2.13)	1.69 (1.25, 2.1)	1.64 (1.25, 2.08)	0.71
Monocyte count (10^9^/L)	0.32 (0.23, 0.51)	0.30 (0.24, 0.5)	0.38 (0.24, 0.52)	0.32 (0.22, 0.52)	0.133
Platelet count (10^9^/L)	194 (161, 235)	194 (160, 232)	197 (165, 240)	191 (159, 237.5)	0.296
SII	475.33 (320.16, 718.08)	480.39 (319.19, 715.71)	465.01 (320.16, 698.31)	481 (329.39, 744.55)	0.771
SIRI	0.84 (0.52, 1.34)	0.82 (0.51, 1.3)	0.88 (0.52, 1.33)	0.84 (0.56, 1.45)	0.295
MPV (fL)	9.9 (9.2, 10.9)	9.8 (9.3, 10.7)	10.4 (9.3, 11.4)	10 (9.1, 10.8)	< 0.001
Thrombocytocrit (L/L)	0.24 (0.2, 0.27)	0.24 (0.21, 0.27)	0.23 (0.19, 0.26)	0.24 (0.19, 0.26)	< 0.001
PDW (CV)	15.9 (13, 16.2)	15.9 (12.94, 16.3)	15.8 (12.6, 16.2)	15.9 (13.5, 16.1)	0.265
HGB (g/L)	128 (118, 139)	126 (118, 137)	131 (122, 142)	125 (116, 138.5)	< 0.001
Total cholesterol (mmol/L)	4.07 (3.31, 5.09)	4.1 (3.39, 5.08)	4.05 (3.25, 5.27)	3.98 (3.23, 5.02)	0.461
Triglyceride (mmol/L)	1.4 (0.98, 2.13)	1.42 (0.99, 2.2)	1.41 (1.02, 2.09)	1.29 (0.9, 2)	0.043
HDL-C (mmol/L)	1.1 (0.91, 1.38)	1.11 (0.92, 1.38)	1.1 (0.92, 1.4)	1.1 (0.91, 1.35)	0.717
LDL-C (mmol/L)	2.33 (1.78, 3.11)	2.37 (1.79, 3.11)	2.32 (1.78, 3.14)	2.32 (1.75, 3.08)	0.855
Fasting blood glucose (mmol/L)	6.15 (5.12, 7.92)	6.15 (5.13, 7.9)	6.07 (5.11, 7.82)	6.27 (5.17, 7.96)	0.703
Lipoprotein (a) (g/L)	209 (115, 352)	200 (117, 374)	192 (98, 331)	237 (138.5, 352)	0.003
Apolipoprotein A1, Median (Q1,Q3)	1.17 (1, 1.37)	1.18 (1, 1.4)	1.17 (1, 1.37)	1.14 (0.99, 1.35)	0.166
Apolipoprotein B, Median (Q1, Q3)	0.81 (0.59, 1.19)	0.82 (0.61, 1.24)	0.81 (0.58, 1.15)	0.79 (0.58, 1.18)	0.429
Cystatin C, Median (Q1, Q3)	0.91 (0.75, 1.15)	0.92 (0.74, 1.15)	0.9 (0.77, 1.09)	0.95 (0.77, 1.21)	0.046
Creatinine, Median (Q1, Q3)	76.4 (61, 90.5)	80 (64, 93.1)	71 (60, 87)	72.2 (59.9, 89.5)	< 0.001
NT- pro BNP, Median (Q1, Q3)	329.8 (168, 749)	336.4 (172, 737.2)	333 (156.4, 772.49)	316 (188, 698.94)	0.117
Uric acid (μmol/L)	342 (274, 425)	345.6 (278, 422)	332.9 (266, 416)	348.9 (274.8, 440)	0.173
Total bilirubin (µmol/L)	11.7 (8.9, 15.3)	11.8 (9.2, 15.6)	11.55 (8.7, 15)	11.5 (8.49, 15.12)	0.112
Direct bilirubin (µmol/L)	3.8 (2.8, 5.4)	3.8 (2.9, 5.3)	3.8 (2.7, 5.5)	3.8 (2.7, 5.5)	0.863
Indirect bilirubin (µmol/L)	7.5 (5.4, 10.2)	7.9 (5.6, 10.5)	7.3 (5.4, 10)	7.0 (4.8, 9.75)	< 0.001
ALT (U/L)	20 (14, 30)	20 (15, 31)	18.4 (14, 28)	20 (14, 30)	0.122
GGT (U/L)	26 (19, 38)	27 (20, 37)	25 (17, 37)	26 (19, 40)	0.241
AST (U/L)	22 (17, 31)	24 (17, 32)	21 (17, 28)	22 (18, 30)	0.005
ALT/AST (U/L)	0.89 (0.67, 1.19)	0.88 (0.64, 1.21)	0.92 (0.68, 1.2)	0.87 (0.67, 1.12)	0.408
Albumin (g/L)	40.1 (37.7, 42.6)	40.2 (37.7, 42.5)	40.1 (37.9, 42.6)	39.8 (37.3, 42.6)	0.388
Total protein, Mean ± SD, (g/L)	66.64 ± 6.06	66.49 ± 5.79	66.97 ± 6.18	66.54 ± 6.47	0.364
Globulin (g/L)	26.5 (23.8, 29.5)	26.2 (23.8, 29.1)	26.6 (23.9, 29.6)	26.7 (23.6, 29.7)	0.163
D-dimer (DDU)	1 (0.38, 2.04)	1.49 (0.7, 2.3)	0.6 (0.28, 1.76)	0.55 (0.3, 1.36)	< 0.001
Fibrinogen (g/L)	2.98 (2.39, 3.69)	2.96 (2.44, 3.58)	2.97 (2.4, 3.74)	3.08 (2.16, 4.01)	0.62
LVEDD (mm)	44 (41, 47)	45 (41, 47)	44 (41, 47)	43 (41, 47)	0.396
LVEF, n (%)	55 (51, 58)	55 (53, 58)	51 (51, 57)	54 (51, 60)	< 0.001
LVPWD (mm)	10 (9, 11)	10 (9, 11)	10 (9, 11)	10 (9, 11)	0.375
IVSD (mm)	10 (9, 11)	10 (9, 11)	10 (9, 11)	10 (9, 11)	0.94
LAD (mm)	38 (35, 41)	38 (35, 41)	38 (35, 42)	38 (35, 42)	0.393
**MACEs, n (%)**	254 (15.22%)	118 (14.3%)	87 (18.39%)	49 (13.21%)	0.067
Cardiac mortality, n (%)	93 (5.57%)	42 (5.09%)	33 (6.98%)	18 (4.85%)	0.286
Unplanned coronary revascularization, n (%)	106 (6.35)	50 (6.06)	38 (8.03)	18 (4.85)	0.152
Heart failure Admission, n (%)	101 (6.05%)	49 (5.94%)	33 (6.98%)	19 (5.12%)	0.523

* Values are given as n (%), Mean ± SD, or median IQR.

BMI, body mass index; CAD, coronary artery disease; PCI, percutaneous coronary intervention; SBP, systolic blood pressure; DBP, diastolic blood pressure; SII, systemic immune-inflammation index; SIRI, systemic inflammatory response index; MPV, mean platelet volume; PDW, platelet distribution width; HGB, hemoglobin; HDL-C, high-density lipoprotein cholesterol; LDL-C, low-density lipoprotein cholesterol; ALT, alanine aminotransferase; AST, aspartate aminotransferase; GGT, gamma-glutamyl transpeptidase; LVEDD, left ventricular end-diastolic dimension; LAD, left atrium diameter; IVSD, interventricular septal thickness at diastole; LVPWD, left ventricular posterior wall in diastole; LVEF, left ventricular ejection fraction; MACEs, major cardiovascular events; AMI, acute myocardial infarction.

### Feature selection and nomogram construction

We performed feature selection using LASSO regression, the Boruta algorithm, and the random forest model, all of which were used to independently screen variables from the same set of 54 candidate predictors. The common variables identified across these methods were subsequently subjected to Cox regression analysis to determine independent prognostic factors for MACEs, and a nomogram was constructed accordingly.

**Figure 1:**
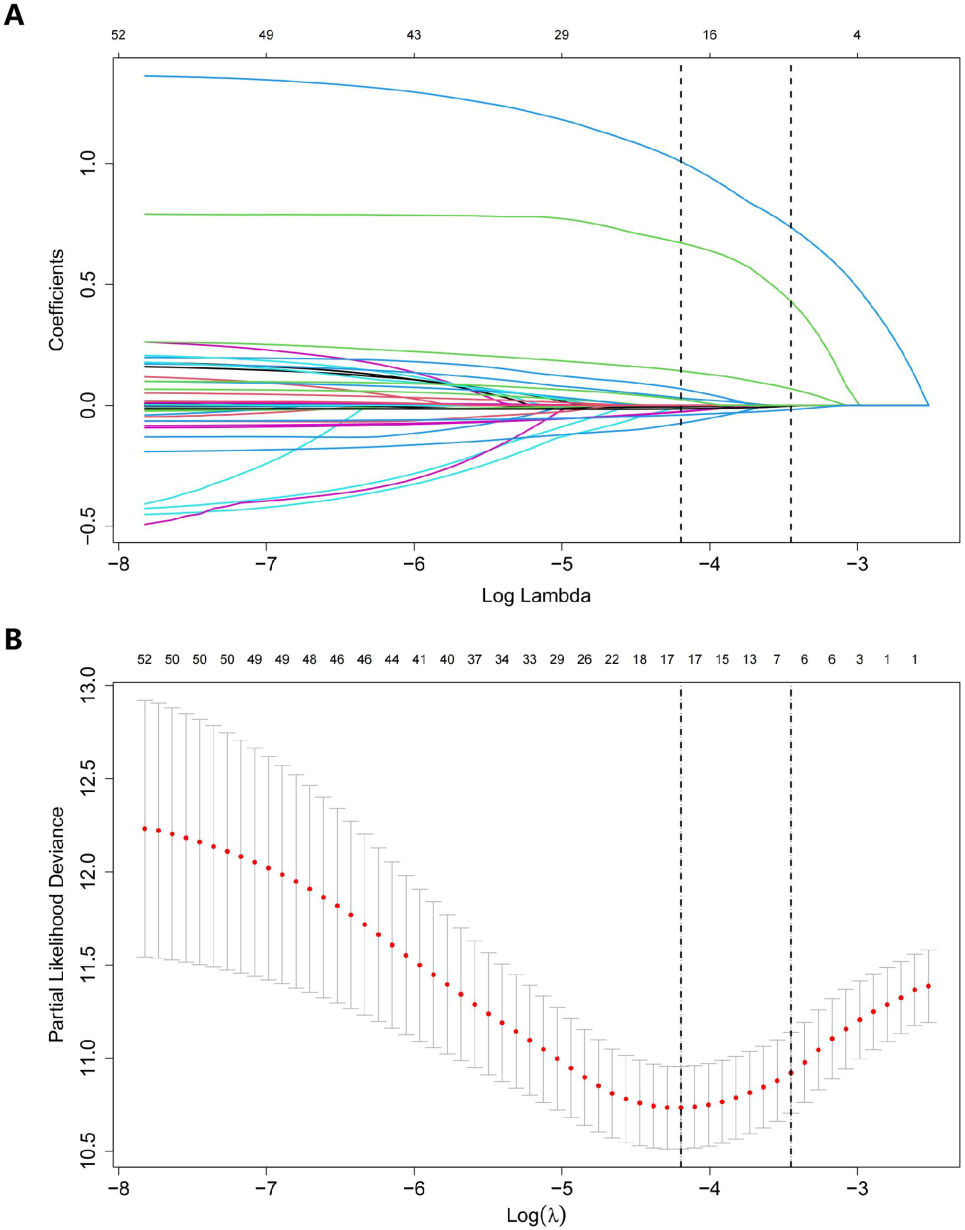
Feature selection using the LASSO regression model. **(A)** LASSO coefficient profiles of the candidate variables. Each curve represents a variable’s coefficient trajectory as the regularization parameter (λ) changes. **(B)** Tuning parameter (λ) selection in the LASSO model via 10-fold cross-validation, and the optimal λ was selected to balance the model complexity and predictive performance.

**Figure 2:**
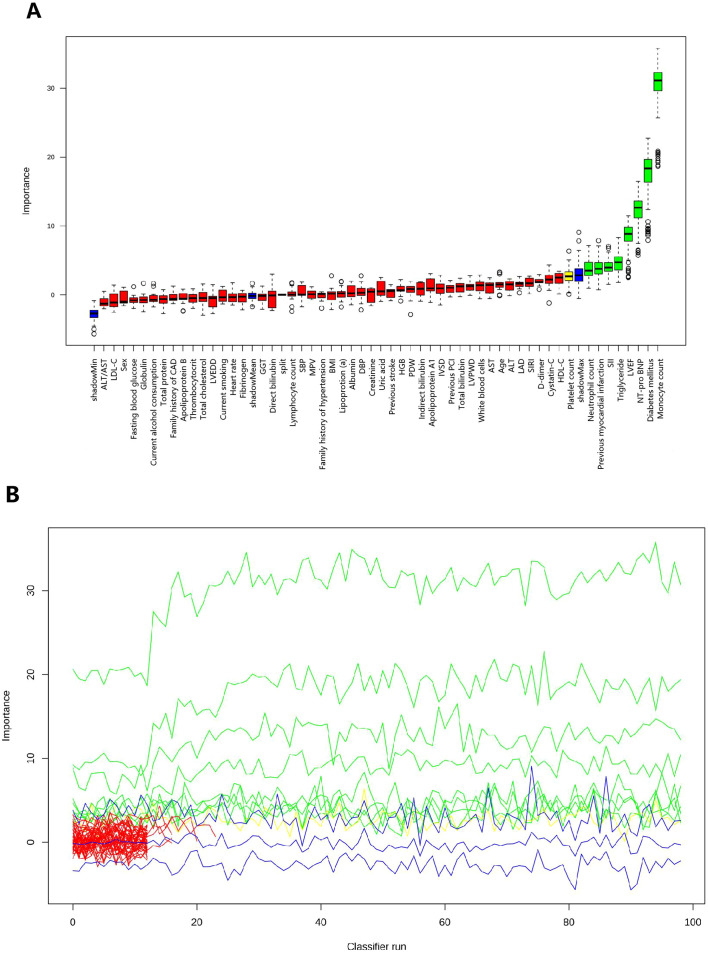
Feature selection using the Boruta algorithm. **(A)** Importance scores of all variables after Boruta selection. The Boruta algorithm selected important features (green), rejected unimportant ones (red), and marked uncertain features as tentative (yellow) using shadow features (blue) as a reference. **(B)** Variation in feature importance over 100 iterations illustrates the consistency of the selected variables.

**Figure 3:**
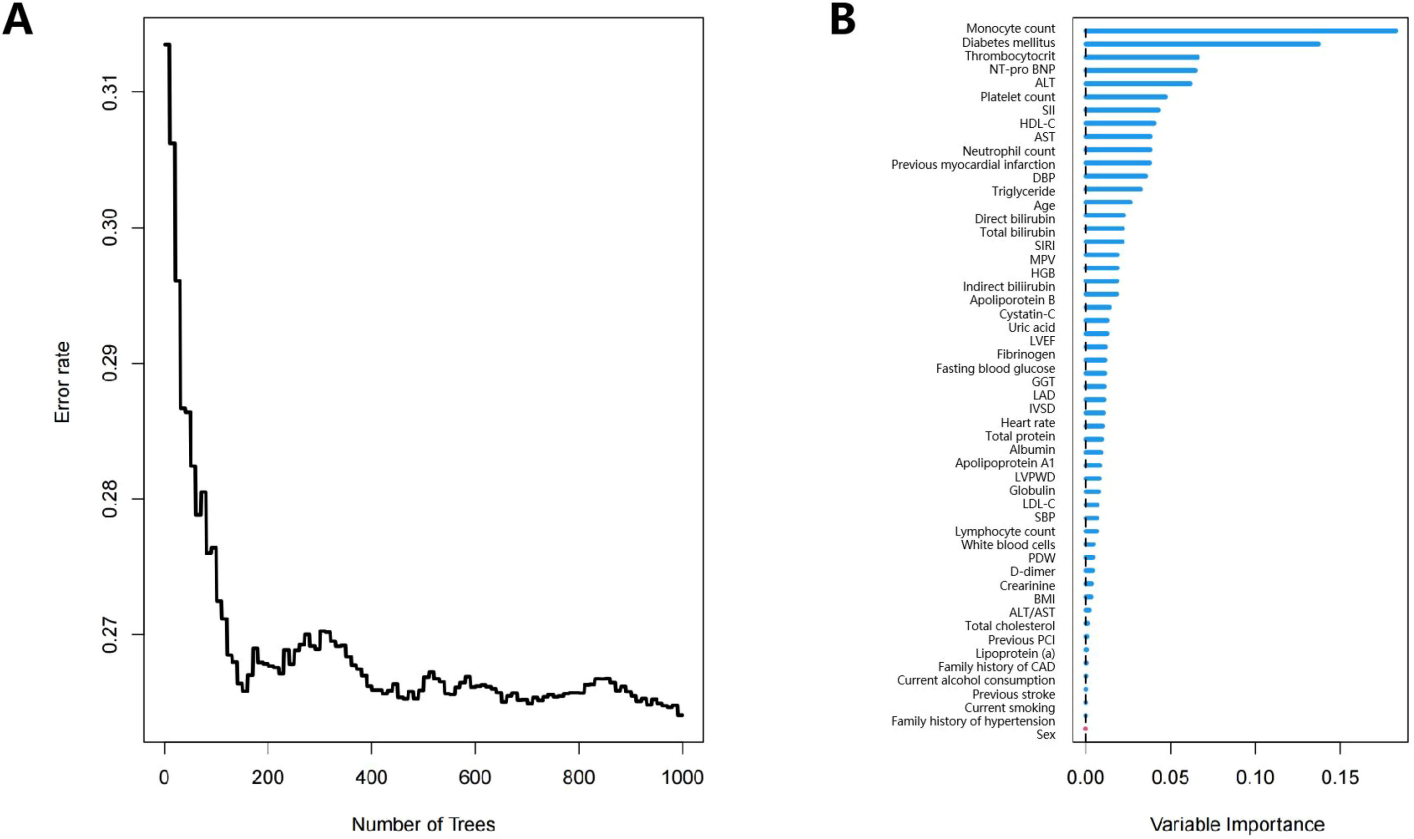
Feature importance ranking and model performance of the random forest classifier. **(A)** The average classification accuracy of the random forest model across different numbers of decision trees illustrating model performance stabilization as tree quantity increases. **(B)** Variable importance ranking based on the mean decrease in accuracy, illustrating the relative contribution of each feature to the classifier’s predictive performance.

**Figure 4:**
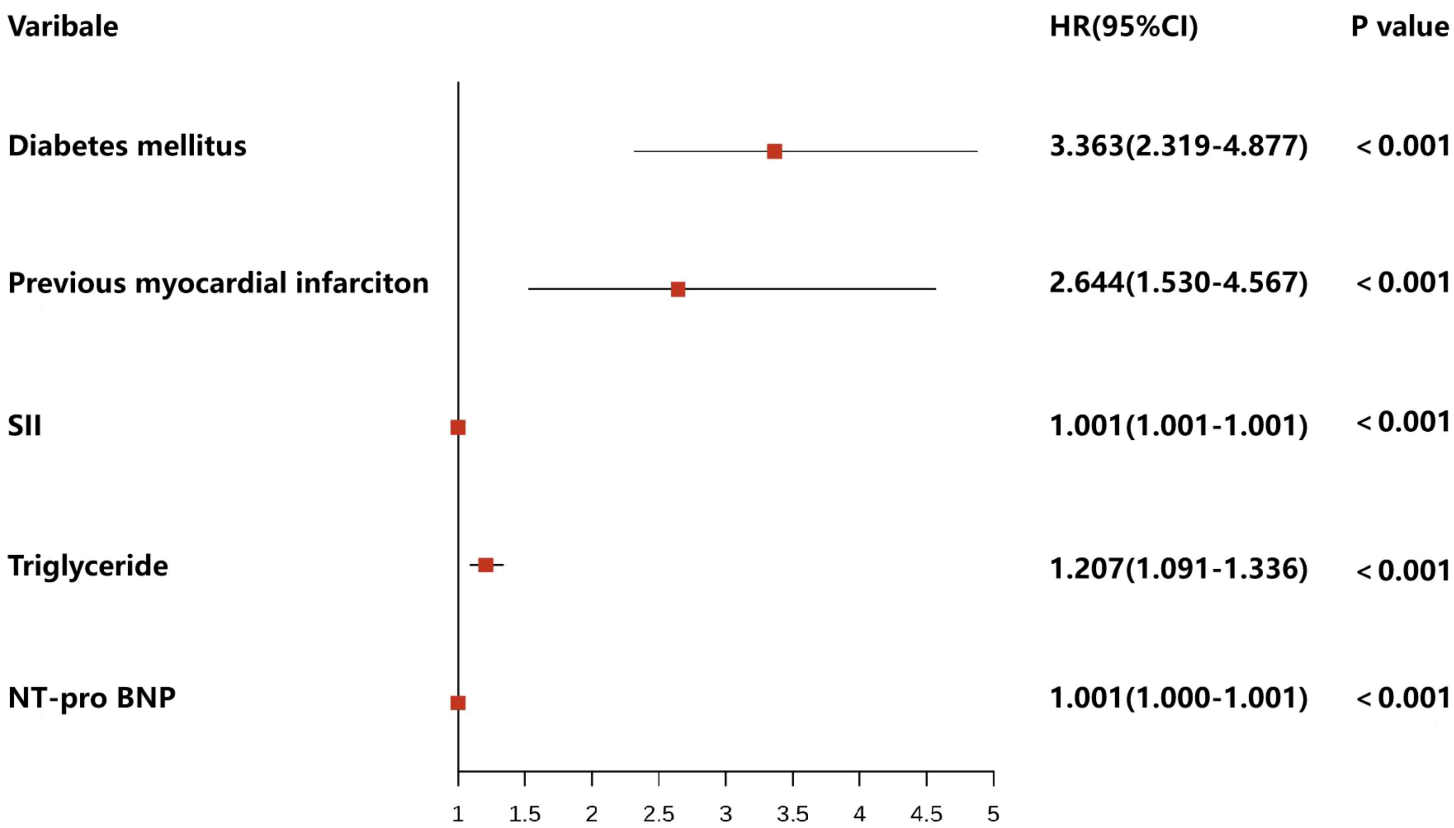
Forest plot of multivariable Cox regression analysis of independent predictors of MACEs.

**Figure 5:**
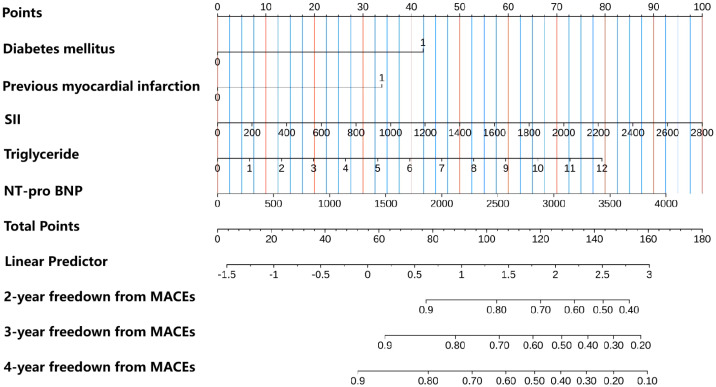
Clinically validated nomogram for predicting the 2-, 3-, and 4-year risk of MACEs in patients with concurrent hypertension, HFpEF, and UAP. The model incorporates five independent prognostic variables identified through machine learning feature selection algorithms: diabetes mellitus (binary), previous myocardial infarction, SII, triglyceride levels, and NT-proBNP (continuous, pg/mL). Risk calculation was performed by drawing a vertical line from each variable value to the corresponding point on the axis, summing the points, and identifying the predicted MACEs probability on the risk axis.

First, LASSO regression was applied to the 54 candidate variables. Using the 1-SE criterion (λ₁SE = 0.03174088), seven predictors were selected: diabetes mellitus, previous myocardial infarction, SII, triglyceride, NT-proBNP, LVEF, and left atrial diameter (LAD) (**Figures 1A, B**). Similarly, the Boruta algorithm independently identified nine relevant features: platelet count, neutrophil count, previous myocardial infarction, SII, triglyceride, LVEF, NT-proBNP, diabetes mellitus, and monocyte count (**Figures 2A, B**). Besides, the random forest model ranked feature importance, and the top 15 variables were selected: monocyte count, diabetes mellitus, thrombocytocrit, NT-proBNP, ALT, platelet count, SII, high-density lipoprotein cholesterol, AST, neutrophil count, previous myocardial infarction, DBP, triglyceride, age, and direct bilirubin (**Figures 3A, B**). To improve the selection of independent prognostic factors, Cox proportional hazards regression analysis was performed on overlapping variables identified by all three methods. Five independent predictors were ultimately determined: Diabetes mellitus (HR = 3.363, 95% CI: 2.31–4.877, *P* < 0.001), previous myocardial infarction (HR = 2.644, 95% CI: 1.53–4.567, *P* < 0.001), SII (HR = 1.001, 95% CI: 1.00–1.001, *P* < 0.001), triglyceride (HR = 1.207, 95% CI: 1.09–1.336, *P* < 0.001), and NT-proBNP (HR = 1.001, 95% CI: 1.000–1.001, *P* < 0.001) (**Figure 4**). Based on the five prognostic factors identified through feature selection and confirmed by Cox regression analysis, a nomogram was developed to provide an individualized risk estimation for MACEs (**Figure 5**).

### Performance of the nomogram

**Figure 6:**
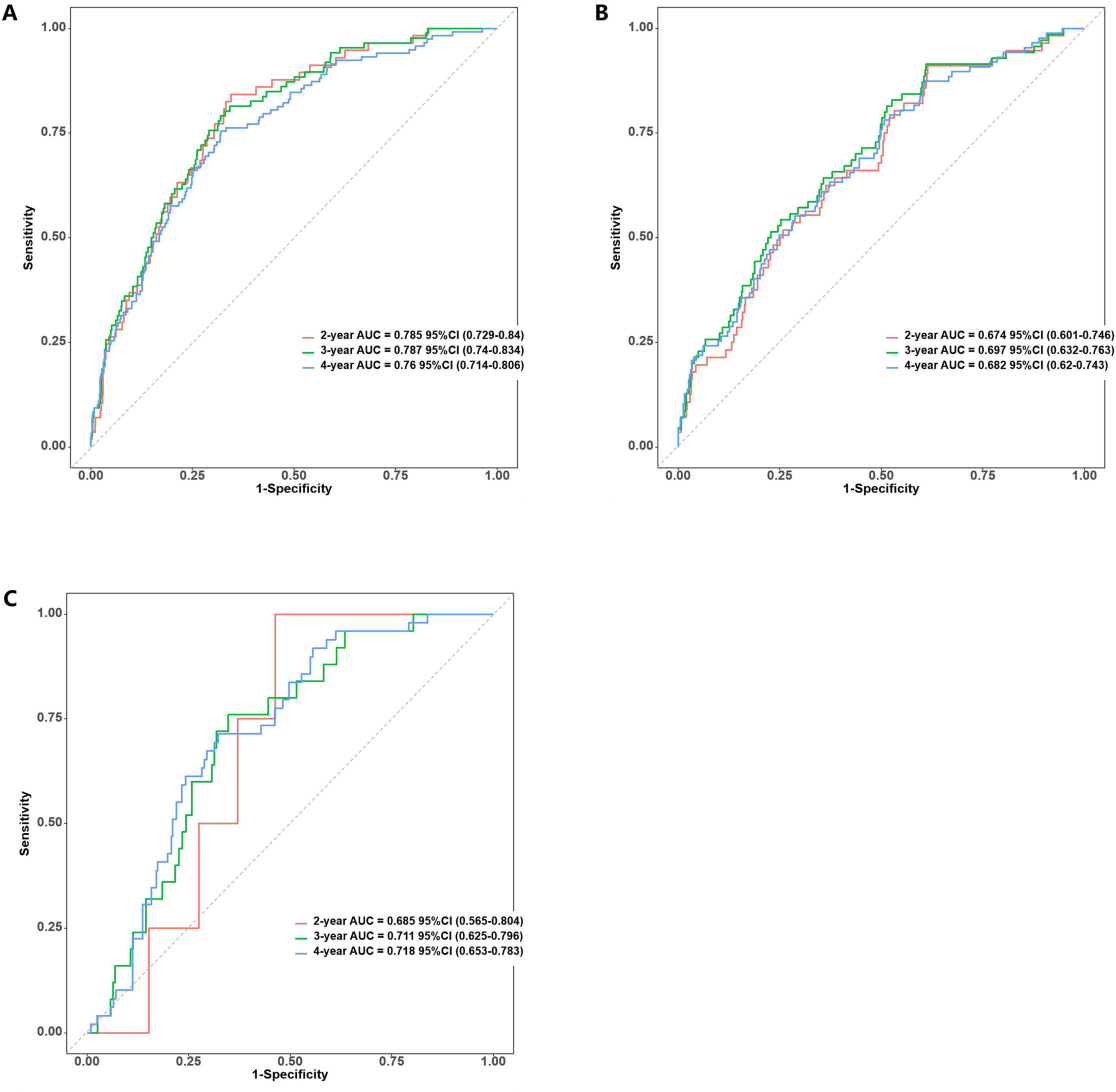
ROC curves of the nomogram for predicting 2-, 3-, and 4-year MACEs in **(A)** development, **(B)** validation, and **(C)** test cohorts. The x-axis represents 1- specificity and the y-axis represents sensitivity.

**Figure 7:**
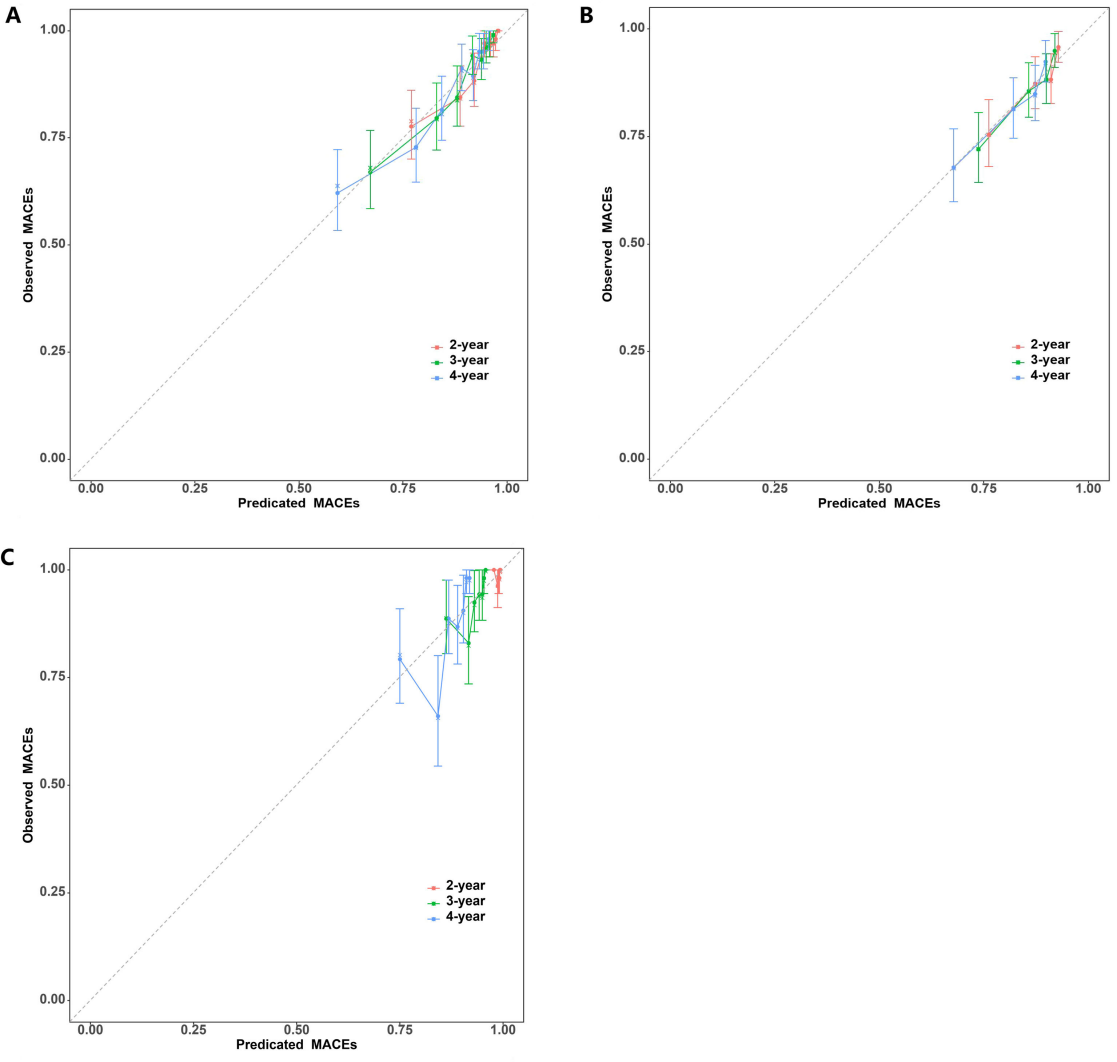
Calibration curves for the nomogram in **(A)** development, **(B)** validation, and **(C)** test cohorts at 2-, 3-, and 4-years. The x-axis represents the nomogram-predicted probability of MACEs, and the y-axis represents the observed MACE proportion. The red, green, and blue symbols correspond to 2-year, 3-year, and 4-year predictions, respectively. Solid lines represent the fitted calibration trends, while the gray dashed line indicates perfect calibration (predicted probability = observed event risk). Points are plotted with vertical error bars denoting 95% confidence intervals. The closer the calibration curves are to the 45° reference line, the better the agreement between predicted and observed risks.

**Figure 8:**
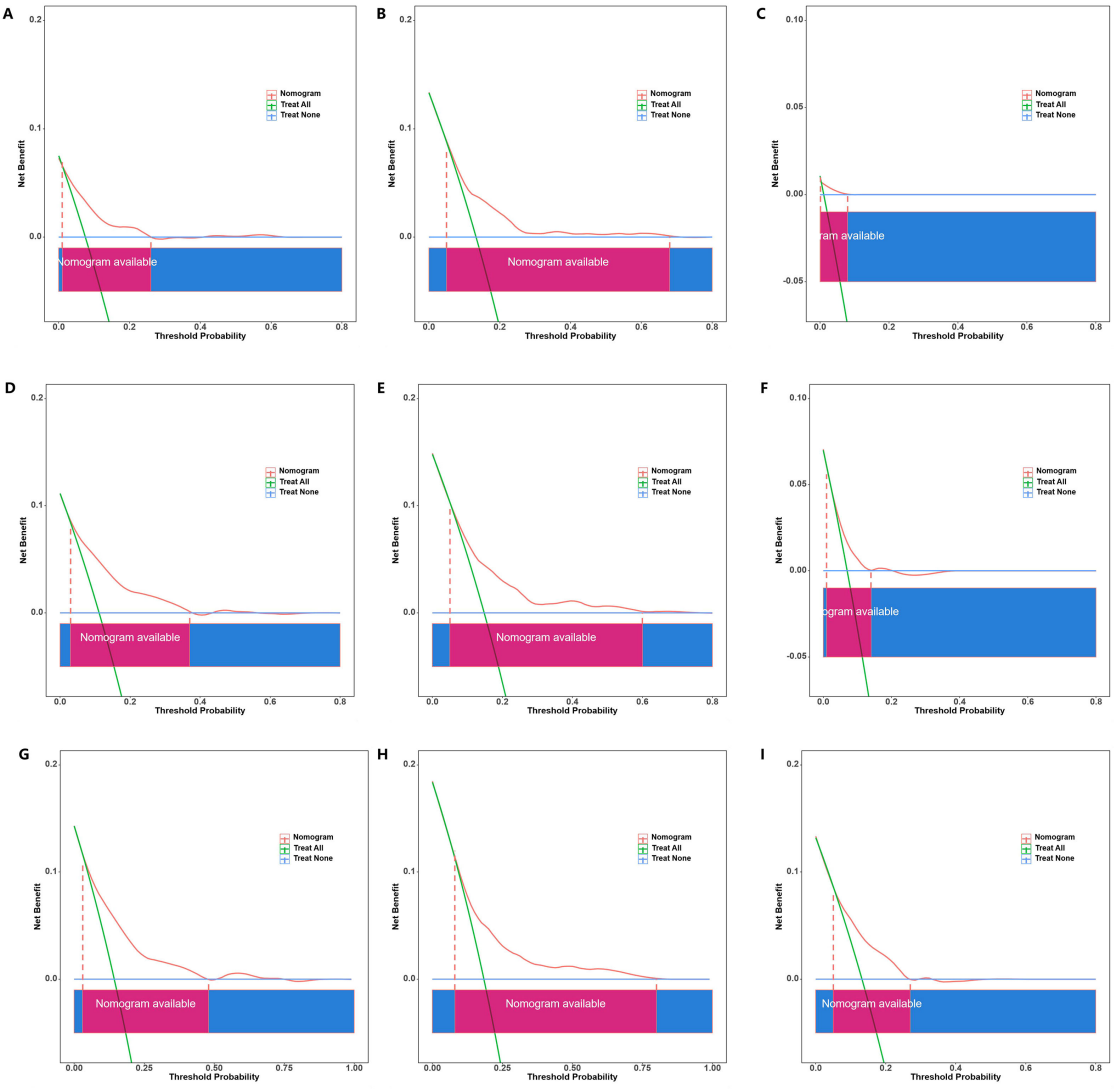
DCA evaluating the clinical utility of the nomogram for predicting 2-, 3-, and 4-year MACEs across different cohorts. DCA for predicting 2-year MACEs in **(A)** development, **(B)** validation, and **(C)** test cohorts. DCA for predicting 3-year MACEs in **(D)** development, **(E)** validation, and **(F)** test cohorts. DCA for predicting 4-year MACEs in the **(G)** development, **(H)** validation, and **(I)** test cohorts. The net benefit of using the nomogram (red line) is plotted against a range of threshold probabilities (x-axis). For comparison, the net benefit of two reference strategies is also shown: treat all (green line) and treat none (blue line). The width and position of the curves indicate the clinical usefulness of the nomogram across different threshold probability ranges; a higher net benefit relative to the treat-all and treat-none strategies suggests better clinical value for decision-making.

**Figure 9:**
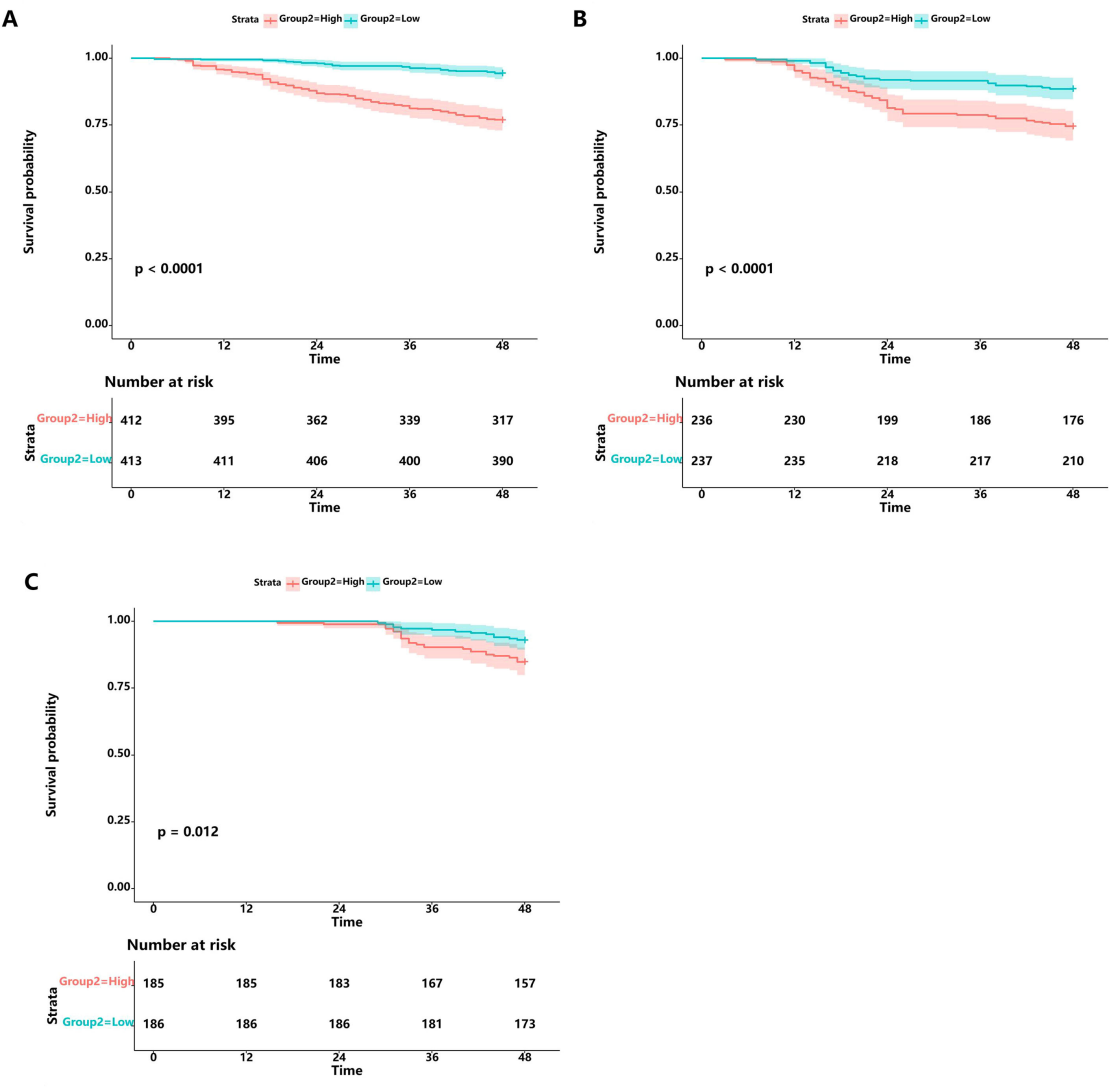
Kaplan–Meier curves illustrating MACEs-free survival stratification based on the machine learning-derived nomogram in patients with concurrent hypertension, HFpEF, and UAP. We divided patients into high-risk and low-risk groups across development **(A)** and validation **(B)** and test **(C)** cohorts.

The predictive performance of the nomogram was comprehensively evaluated using ROC curves, TIME-AUC, calibration curves, DCA, and Kaplan–Meier survival analysis. In the development cohort, the nomogram demonstrated AUCs of 0.785 (95% CI: 0.729-0.840), 0.787 (95% CI: 0.740-0.834), and 0.760 95%(0.714-0.806) for predicting 2-,3-,and 4-year MACEs, respectively, indicating acceptable discrimination (**Figure 6A**). In the validation cohort, AUCs were 0.674 (95% CI: 0.601-0.746), 0.697 95CI(0.632-0.763), and 0.682 (95% CI: 0.620-0.743) for 2-, 3-, and 4-year predictions, respectively, reflecting relatively weak discrimination (**Figure 6B**). In the test cohort, AUCs were 0.685 (95% CI: 0.565-0.804), 0.711 (95% CI: 0.625-0.796), and 0.718 (95% CI: 0.653-0.783) for 2-, 3-, and 4-year predictions, respectively, corresponding to poor (2-year) and acceptable (3- and 4-year) discrimination (**Figure 6C**). Furthermore, the TIME-AUC analysis confirmed the stable predictive performance of the nonogram throughout the follow-up period, reinforcing its long-term prognostic value (**Supplementary Figures 1A–C**). Calibration curve analysis showed generally acceptable agreement between predicted and observed MACE risks, supporting the calibration of the nomogram across the development, validation, and test cohorts. (**Figures 7A–C**). DCA provided further insights into the clinical utility of our nomogram. The optimal decision thresholds for predicting MACEs in the training, validation, and test cohorts were 1%**–**28%, 5%**–**70%, and 1%**–**9% (2-year), 1%**–**38%, 5%**–**60%, and 1%**–**15% (3-year), and 3%**–**48%, 13%**–**81%, and 5%**–**28% (4-year), respectively (**Figures 8A–I**). These results indicate that the nomogram offers substantial clinical net benefits over a wide range of decision thresholds, emphasizing its role as a valuable tool for individualized risk stratification and clinical decision-making. Moreover, the clinical benefit of the nomogram was compared with that of a single predictor. The results revealed that the nomogram demonstrated a higher net benefit at all time points, indicating that integrating multiple key predictors was superior to any single variable, further highlighting its clinical value in precision medicine (**Supplementary Figures 2A–I**). Moreover, risk scores were calculated for each patient, and participants in all cohorts were categorized into high- and low-risk score groups. Kaplan–Meier survival analysis revealed that the incidence of MACEs was significantly higher in the high-risk group than in the low-risk group across all cohorts (*P* < 0.05), thereby validating the predictive power and clinical applicability of the nomogram (**Figures 9A–C**).

Furthermore, we developed an interactive, web-based, dynamic nomogram model using the CASCADE platform, enabling real-time, individualized risk assessment for MACEs (**Supplementary Figure 3**). By simply inputting the five key clinical variables identified in this study, clinicians can instantly obtain a personalized risk prediction for each patient. This tool facilitates more precise and individualized clinical decision-making, improving the early identification of high-risk patients and optimizing their follow-up management strategies.

## Discussion

In this study, the prognostic value of metabolic and inflammatory biomarkers was systematically explored in patients with coexisting hypertension, HFpEF, and UAP. For the first time, a multidimensional risk assessment model was developed, integrating inflammation, metabolism, and cardiac function. Using machine learning methodologies, including LASSO regression, the Boruta algorithm, and random forest analysis, five key independent predictors of MACEs were identified: Diabetes mellitus, previous myocardial infarction, SII, triglyceride, and NT-proBNP. On the basis of these findings, we developed and validated a clinically accessible prediction model that demonstrated acceptable discrimination, generally good calibration, and potential decision-making utility, although its performance was more modest in the validation and test cohorts than in the development cohort. Furthermore, we developed a dynamic, web-based nomogram using the CASCADE platform, enabling real-time, individualized risk prediction and improving clinical decision-making.

Contemporary cardiovascular medicine is witnessing a paradigm shift from reductionist, single-disease models to integrative, systems-level frameworks of disease conceptualization (4, 6). Within this evolving landscape, the concurrent presence of hypertension, HFpEF, and UAP, particularly prevalent among elderly populations and individuals with severe metabolic dysfunction, represents more than simple disease coexistence. This cardiovascular triad is a unique pathophysiological entity characterized by complex bidirectional relationships among the vascular, myocardial, and coronary subsystems. Rather than representing independent, parallel disease processes, patients with concurrent hypertension, HFpEF, and UAP manifests as an intricate pathobiological network where inflammation, metabolic dysregulation, and hemodynamic perturbations engage in continuous dynamic cross-talk. This multimorbid phenotype exemplifies what might be considered a “cardiovascular system failure syndrome,” wherein initial pathological alterations in one domain trigger a cascade of maladaptive responses across interconnected biological systems. Within this framework, cardiovascular risk emerges not from the arithmetic sum of individual diseases but from the nonlinear interactions between their underlying pathophysiological mechanisms, fundamentally restructuring the risk architecture of affected individuals. Conventional risk stratification tools, predominantly calibrated for discrete cardiovascular conditions, systematically misrepresent event probabilities in this population by failing to capture these emergent synergistic interactions. This limitation is addressed by our integrated biomarker-driven modeling approach, which identifies key prognostic signals that traverse traditional disease boundaries. This methodology enhances risk discrimination and potentially illuminates critical nodes within the pathophysiological network where targeted interventions could simultaneously modulate multiple disease pathways. Ultimately, this systems-oriented paradigm provides both conceptual and practical frameworks for transitioning from fragmented, disease-centric management to integrated, mechanism-oriented therapeutic strategies tailored to the biological complexity of this increasingly prevalent comorbidity of cardiovascular diseases.

In our previous study, we investigated the integration of multivariate clinical data with machine-learning techniques to develop prognostic models for cardiovascular event prediction (6, 17–24). The present study extends this prior work by focusing specifically on the clinically important subgroup of patients with concomitant hypertension. This distinction is relevant because hypertension is highly prevalent in HFpEF and is mechanistically linked to arterial stiffness, endothelial dysfunction, systemic inflammation, ventricular-vascular uncoupling, and increased myocardial oxygen demand, all of which may contribute to adverse cardiovascular outcomes in patients with coexisting UA and HFpEF. Rather than proposing a new disease entity, the present study highlights a common high-risk comorbidity pattern in which individualized risk stratification remains challenging. Although established guidelines provide disease-specific recommendations for hypertension, HFpEF, and UAP, respectively, the complexity arising from their concurrent presentation is not fully addressed in contemporary clinical practice Current care pathways often focus on individual diseases, which may lead to fragmented risk assessment and insufficient consideration of the combined metabolic, inflammatory, ischemic, and hemodynamic burden in these patients. For example, HFpEF management emphasizes symptom relief, volume optimization, and control of contributing comorbidities (2), whereas UAP requires timely ischemic risk assessment and intensive secondary prevention (3), and hypertension management prioritizes blood pressure control and global cardiovascular risk reduction (1). When these conditions coexist, clinicians must integrate these overlapping but distinct therapeutic priorities while accounting for the patient’s overall risk profile. Therefore, although previous studies have examined isolated conditions or pairwise disease combinations, evidence remains limited regarding integrated long-term risk stratification in patients with concurrent hypertension, HFpEF, and UAP. This gap supports the need for a dedicated prognostic model tailored to this frequently encountered but clinically complex population.

Conventional prediction models relying on linear statistical methodologies inadequately capture the complex nonlinear interactions between inflammatory cascades and metabolic dysregulation that define this multimorbid phenotype. To address these limitations, we used machine learning strategies, including LASSO regression, Boruta feature selection algorithms, and random forest modeling to identify key predictive signatures from multimodal variables in a large, real-world cohort of patients with concurrent hypertension, HFpEF, and unstable angina. Our approach outperforms traditional prediction models that focus exclusively on single-disease endpoints by highlighting disease-disease interaction patterns and their prognostic implications. The integrated risk prediction tool showed potential for supporting earlier recognition of high-risk patients and individualized clinical assessment, but its generalizability and impact on targeted intervention strategies require confirmation in prospective studies.

Our machine learning-derived model identified several independent predictors, with SII emerging as a significant and novel prognostic indicator. SII has been recognized as an important biomarker across the cardiovascular disease spectrum (25–27). In hypertensive cohorts, elevated SII demonstrates significant associations with all-cause mortality, reflecting the deleterious long-term consequences of chronic low-grade inflammation on cardiovascular pathophysiology (25). Moreover, SII has been independently linked to heart failure progression (26) and outperforms traditional risk factors in predicting MACEs among patients with coronary artery disease (27). Our findings extend these observations, suggesting that SII may function as an inflammatory marker and a central pathophysiological mediator in the complex interplay between hypertension, HFpEF, and UAP. The additional predictors identified through our feature selection algorithms represent complementary pathophysiological domains relevant to the patients with concurrent hypertension, HFpEF, and UAP. Diabetes mellitus contributes to adverse cardiac structural and functional remodeling through multiple mechanisms beyond dysglycemia, including insulin resistance and pro-inflammatory signaling cascades (28). Previous myocardial infarction indicates not only anatomical high-risk coronary features but also potential myocardial substrate abnormalities characterized by fibrosis and electrical remodeling, processes that significantly increase the vulnerability to subsequent cardiovascular events (29). Elevated triglyceride levels indicate disturbances in lipid metabolism that may exacerbate endothelial dysfunction and promote atherosclerotic plaque instability (30). NT-proBNP, a well-established marker of myocardial wall stress, accurately captures the hemodynamic perturbations and diastolic dysfunction that characterize this patient population (31).

Despite being individually significant, these predictors synergistically interacted within our integrated risk model. Using high-dimensional feature screening and ensemble machine learning techniques, we developed a prediction framework with enhanced accuracy and generalizability than conventional approaches. In addition to improving individual patient management, CASCADE provides a potential quality metric for healthcare systems, facilitating a shift from isolated, single-disease performance indicators to multidimensional quality assessment frameworks. This transformation is consistent with value-based care models because it enables a comprehensive evaluation of clinical outcomes across traditional disease boundaries. The platform’s ability to capture the complex interactions between hypertension, HFpEF, and UAP introduces a novel paradigm for precision quantification in cardiovascular medicine. Notably, CASCADE provides a scalable and reproducible framework to address the persistent challenge of “multi-disease co-management,” which has consistently impeded optimal care delivery for patients with complex cardiovascular conditions. CASCADE effectively bridges the implementation gap between sophisticated risk prediction science and routine clinical practice by translating advanced machine learning insights into a user-friendly clinical decision support tool.

### Study limitations

Despite the strengths of this study, there are several limitations. First, the retrospective nature of this study may lead to potential selection bias and residual confounding despite efforts to ensure data integrity and cohort balance. Second, patients were recruited from a limited source population, and baseline characteristics, diagnostic practices, treatment patterns, and laboratory measurement protocols may differ across regions and institutions. Therefore, the generalizability of the nomogram to other populations-such as different ethnic groups, healthcare systems, and patient spectra of hypertension, HFpEF, and UAP-may be limited. Third, although missing data handling was performed via exclusion of patients with incomplete clinical or laboratory variables, complete-case analysis may reduce representativeness. Fourth, treatment effects after enrollment were not fully captured due to variability in real-world management and the retrospective setting. Fifth, although all included patients underwent coronary angiography during the index hospitalization and none underwent index revascularization, detailed angiographic characteristics, including lesion location, stenosis severity, plaque morphology, and coronary disease burden, were not systematically incorporated into the prediction model. Therefore, the potential influence of baseline coronary anatomical complexity on long-term outcomes could not be fully assessed. Future studies integrating angiographic and functional coronary assessment may further improve risk stratification in patients with concurrent hypertension, HFpEF, and unstable angina. Finally, although the nomogram was validated internally using development, validation, and test cohorts, prospective and external validation is still necessary to confirm the model’s transportability, calibration, and clinical utility in independent populations and prospective clinical workflows.

## Conclusions

In this multicenter CASCADE cohort study, a unique cardiovascular phenotype characterized by the pathophysiological convergence of hypertension, HFpEF, and UAP was described. Our predictive model, developed using machine learning techniques and integrating traditional risk factors and novel inflammatory-metabolic biomarkers, exhibited good performance prediction and consistent calibration across geographically diverse validation cohorts.

## Data Availability

Individual participant data that underlie the results reported in this article, after de-identification can be obtained from the corresponding author upon reasonable request.
