# Supplementary material for: Risk prediction for long-term cardiovascular events in patients with concurrent hypertension, HFpEF, and unstable angina: a multicenter machine learning-assisted cohort study
Source: Front Endocrinol (Lausanne). 2026 Jul 23;17:1851201. doi: 10.3389/fendo.2026.1851201 (PMC13441924; doi:10.3389/fendo.2026.1851201)
Supplement: Supplementary file 1 [file SupplementaryFile1.docx]

**Supplemental Table 1. Inclusion and exclusion criteria.**

**Inclusion criteria**

Hypertension,

Heart failure with preserved ejection fraction,

Unstable angina pectoris,

Age ≥18 years.

**Exclusion criteria**

Pacemaker implantation,

Malignancy,

Prior coronary artery bypass grafting,

Combined severe heart valve disease,

Severe infection or inflammation,

Severe renal insufficiency (eGFR < 30 mL/min/1.73m²),

Poor compliance to treatment

Incomplete clinical or laboratory data for clinical variable

Lost to follow-up.

**Supplemental Figure 1. The time-dependent AUC curves of the prediction model display the time-dependent AUC values for development (A) and validation (B) and test (C) cohorts across various time points.**

**Supplemental Figure 2. Decision curve analysis was performed to assess the predictive nomogram and five univariate individual models at 2-, 3-, and 4-year intervals across the development cohort (A, D, and G), the validation cohort (B, E, and H), and the test cohort (C, F, and I).**

**Supplemental Figure 3. The CASCADE web-based clinical decision support platform operationalizes our validated prediction nomogram for patients with the cardiovascular triad syndrome. This interactive interface enables real-time risk stratification at the point of care through a user-friendly design optimized for clinical workflow integration. Clinicians input five evidence-based prognostic variables: diabetes mellitus status, previous myocardial infarction history, systemic immune-inflammation index (SII), triglyceride levels, and NT-proBNP concentrations. The platform instantly generates individualized 2-, 3-, and 4-year MACE probability estimates with risk categorization.**
